# Treatment of metabolic acidosis with sodium bicarbonate delays progression of chronic kidney disease: the UBI Study

**DOI:** 10.1007/s40620-019-00656-5

**Published:** 2019-10-09

**Authors:** Biagio R. Di Iorio, Antonio Bellasi, Kalani L. Raphael, Domenico Santoro, Filippo Aucella, Luciano Garofano, Michele Ceccarelli, Luca Di Lullo, Giovanna Capolongo, Mattia Di Iorio, Pasquale Guastaferro, Giovambattista Capasso, The UBI Study Group, Vincenzo Barbera, Annamaria Bruzzese, Valeria Canale, Giuseppe Conte, Vincenzo Crozza, Adamasco Cupisti, Antonella De Blasio, Emanuele De Simone, Lucia Di Micco, Fulvio Fiorini, Rachele Grifa, Raffaella Nardone, Matteo Piemontese, Maria Luisa Sirico, Fabio Vitale

**Affiliations:** 1 Nephrology and Dialysis Unit, PO “A. Landolfi”, Solofra, Avellino Italy; 2 Department of Research, Innovation, Brand Reputation, Bergamo Hospital, ASST Papa Giovanni XXIII, Bergamo, Italy; 3 Division of Nephrology and Hypertension, Department of Internal Medicine, University of Utah Health, Salt Lake City, UT USA; 4 Dialysis and Nephrology Unit, University of Messina, Messina, Italy; 5 Department of Nephrology and Dialysis, IRCCS “Casa Sollievo della Sofferenza”, San Giovanni Rotondo, Foggia, Italy; 6 Biogem, Section of Genetic and Translational Medicine, Ariano Irpino, Avellino Italy; 7 Department of Science and Technology, University of Sannio, Benevento, Italy; 8 Department of Nephrology and Dialysis, “L. Parodi-Delfino” Hospital, Colleferro, Roma, Italy; 9 Department of Translational Medical Sciences, University of Campania “Luigi Vanvitelli”, Naples, Italy; 10 Data Scientist, Landolfi Nephrology Dialysis Consultant, Solofra, Avellino, Italy; 11 Department of Nephrology, “G. Criscuoli” Hospital, Sant’Angelo dei Lombardi, Avellino Italia; 12 Nephrology and Dialysis, “Antonio Cardarelli” hospital, Naples, Italia

**Keywords:** Metabolic acidosis, Chronic kidney disease, Sodium bicarbonate

## Abstract

**Background:**

Metabolic acidosis is associated with accelerated progression of chronic kidney disease (CKD). Whether treatment of metabolic acidosis with sodium bicarbonate improves kidney and patient survival in CKD is unclear.

**Methods:**

We conducted a randomized (ratio 1:1). open-label, controlled trial (NCT number: NCT01640119. www.clinicaltrials.gov) to determine the effect in patients with CKD stage 3–5 of treatment of metabolic acidosis with sodium bicarbonate (SB) on creatinine doubling (primary endpoint), all-cause mortality and time to renal replacement therapy compared to standard care (SC) over 36-months. Parametric, non-parametric tests and survival analyses were used to assess the effect of SB on these outcomes.

**Results:**

A total of 376 and 364 individuals with mean (SD) age 67.8 (14.9) years, creatinine clearance 30 (12) ml/min, and serum bicarbonate 21.5 (2.4) mmol/l were enrolled in SB and SC, respectively. Mean (SD) follow-up was 29.6 (9.8) vs 30.3 (10.7) months in SC and SB. respectively. The mean (SD) daily doses of SB was 1.13 (0.10). 1.12 (0.11). and 1.09 (0.12) mmol/kg*bw/day in the first, second and third year of follow-up, respectively. A total of 87 participants reached the primary endpoint [62 (17.0%) in SC vs 25 (6.6%) in SB, p < 0.001). Similarly, 71 participants [45 (12.3%) in SC and 26 (6.9%) in SB, p = 0.016] started dialysis while 37 participants [25 (6.8%) in SC and 12 (3.1%) in SB, p = 0.004] died. There were no significant effect of SB on blood pressure, total body weight or hospitalizations.

**Conclusion:**

In persons with CKD 3–5 without advanced stages of chronic heart failure, treatment of metabolic acidosis with sodium bicarbonate is safe and improves kidney and patient survival.

**Electronic supplementary material:**

The online version of this article (10.1007/s40620-019-00656-5) contains supplementary material, which is available to authorized users.

## Introduction

One of the most important kidney functions is to maintain normal acid–base balance by eliminating non-volatile acids, primarily as ammonium, and reabsorbing and generating bicarbonate. When chronic kidney disease (CKD) develops, acid excretion is impaired leading to acid retention and metabolic acidosis, commonly defined as a serum bicarbonate concentration < 22 mmol/l [1, 2]. Metabolic acidosis is present in approximately 15% of patients with CKD [1] and is associated with several serious consequences including risk of CKD progression, skeletal muscle catabolism, insulin resistance, bone demineralization, and mortality [3, 4]. To mitigate these adverse consequences, clinical practice guidelines recommend treating metabolic acidosis with base supplementation [2]. However, evidence from well-designed, large, multicenter clinical trials that treatment of metabolic acidosis with base improves bone, muscle, and kidney health, or patient survival is lacking [5–9].

The Use of Bicarbonate in Chronic Renal Insufficiency (UBI) study was conducted to determine whether treatment of metabolic acidosis with alkali supplementation using oral sodium bicarbonate in patients with CKD preserves kidney function [10]. Secondary outcomes included all-cause mortality and initiation of renal replacement therapy [10]. We herein report the primary and secondary endpoint results of the largest randomized controlled study to determine the effects of treatment of metabolic acidosis on kidney and patient survival in patients with CKD stage 3–5 patients [10].

## Methods

### Trial design and oversight

The UBI Study was a multicenter, randomized, unblinded, pragmatic controlled trial. Patients at 10 Nephrology units in Italy with CKD stage 3–5 and metabolic acidosis were enrolled from January 2013 through September 2014 and randomized (ratio: 1:1) to receive sodium bicarbonate (SB) or standard care (SC) for 36 months, the occurrence of a study endpoint (whichever comes first) or lost to follow-up. To ensure allocation concealment, randomization was centralized [10].

The study protocol was approved by the Campania Nord Ethic Committee, registered on (NCT01640119) and conducted according to the principles embodied in the Declaration of Helsinki and Good Clinical Practice. All participants signed an informed consent document.

### Eligibility

Inclusion criteria were serum bicarbonate > 18 and < 24 mmol/l, age > 18 years and CKD stage 3–5. Exclusion criteria were active cancer, active autoimmune disease, New York Heart Association class III or IV heart failure symptoms, blood pressure (BP) > 150/90 mmHg, limb amputation, history of cerebrovascular disease, neurological bladder or ureterosigmoidostomy, or current use of calcium carbonate.

### Intervention

SB was administered twice daily to achieve a target serum bicarbonate concentration of 24–28 mmol/l in the experimental group. The starting daily dose was calculated to replace half of the bicarbonate deficit (bicarbonate deficit in mmol = [24 − serum bicarbonate in mmol/l] × [total body weight (kg) × 0.5]). Of note, the dosage of SB is expressed as mmol/kg of ideal body weight/day (conversion factor: 1 mmol = 84 mg of SB and 23 mg elemental sodium). The daily dose was subsequently increased by 25% every week until the serum bicarbonate target was achieved. The dose of SB was adjusted to keep serum bicarbonate levels < 28 mmol/l as necessary. Serum bicarbonate concentration was measured weekly for the first 3 months and every 3 months thereafter. In the SC group, rescue treatment with SB was not protocolized and was permissible for safety reasons at the attending physicianʻs discretion. Similarly, diet was not protocolized either and participants received nutritional counseling and were prescribed a low protein (0.8 or 0.6 or 0.3 g/kg/day, depending on CKD severity), low phosphorus (< 800 mg/day) and controlled sodium (< 7 g/day) diet at the attending physicianʻs discretion [10]. Medical management to achieve suggested targets for glycated hemoglobin, bone mineral metabolism, BP, anemia, iron status, and cholesterol was left to the discretion of the participant’s attending physician. The targets were: BP < 135/90 mmHg, serum hemoglobin 11–12 g/dl, low density lipoprotein (LDL) cholesterol < 100 mg/dl, serum phosphorous 2.7–4.6 mg/dl, serum calcium 8.4–10.2 mg/dl, serum parathyroid hormone (PTH) 70–150 pg/ml and serum 25 (OH) vitamin D 30–100 nmol/l [2].

### Study endpoints

The primary outcome was time to doubling of serum creatinine. All-cause mortality and time to initiation of renal replacement therapy were secondary endpoints. The effect of SB on blood pressure, nutritional parameters, fluid retention, and hospitalizations was also evaluated as safety endpoints. Primary and secondary efficacy study endpoints were adjudicated by a physician blinded to the participant’s treatment assignment while safety endpoints were adjudicated by the attending physician.

### Study procedures

After the first 3 months, anthropometric, vital signs, and creatinine clearance measured via 24-h urine collection were measured at least every 3 months, and according to the standard practice at the study center where the participant was followed. Other laboratory parameters [serum levels of albumin, C-reactive protein (CRP), sodium, potassium, chloride, homocysteine, lipids, calcium, phosphorus, PTH, alkaline phosphatase, transferrin saturation (TSAT), ferritin, creatinine, urea, and blood cell count; and 24-h urine urea, sodium, protein, creatinine, phosphate] were tested at baseline and every 6 months or according to the standard practice at the study center where the participant was followed. Non-study medications (particularly, antihypertensive and diuretic medications) were recorded at baseline and every 6 months. All laboratory measurements were performed at the local laboratory as per standard of care.

Renal function was assessed by 24-h creatinine clearance, which is the standard of care in Italy. However, estimated glomerular filtration rate (eGFR) was calculated using the modification of diet in renal disease (MDRD) formula [11]. Protein intake was calculated using the Maroni formula [12]. The study visit schedule as well as the frequency of renal function assessment was decided by the attending physician based on the degree of renal function impairment and according to the center standard of care.

### Sample size calculation

Assuming a primary endpoint occurrence rate of 8.7% and 16.9% among treated and untreated subjects, a two-tailed statistical test with type I error of 5%, and 10% attrition, it was determined that 364 subjects per arm would provide 80% power for the primary endpoint.

### Statistical analysis

Continuous variables are presented as mean ± standard deviation (SD) or median (interquartile range) as appropriate. Categorical variables are presented as proportions. Parametric (t test) and non-parametric tests (Wilcoxon sum rank test, Chi square test, Fisher exact test) were used as appropriate to evaluate for between- and within-group differences according to the treatment assignment. CKD stage was defined according to the estimated glomerular filtration rate (eGFR) [11].

Univariable- and multivariable-adjusted survival analysis were performed. By design, all patients were followed until the occurrence of a study endpoint (i.e. patients who experienced a doubling of creatinine were no longer followed for dialysis initiation or death and patients who initiated dialysis were no longer followed for death) or study completion (36 months of follow-up). First, the cumulative incidence of the study endpoints (creatinine doubling, dialysis, death) by treatment assignment were constructed by the Kaplan–Meier method and the log rank test was used to determine statistical significance. Cox proportional hazard regression analyses were applied to estimate the hazard ratios (HR) of SB relative to SC for the three endpoints (creatinine doubling, dialysis, death). The Cox proportional hazard assumption was verified in the unadjusted models by using the Schoenfeld residuals against the transformed time method. Cox proportional hazard regression analysis are presented as: (1) unadjusted; (2) adjusted for age and sex (model 1); (3) adjusted for model 1 and body mass index (BMI), systolic and diastolic BP (model 2); (4) adjusted for model 2 and baseline creatinine clearance, cardiovascular disease (CVD), diabetes, hypertension (model 3); (5) adjusted for model 3 and proteinuria and use of medications that inhibit the renin–angiotensin–aldosterone system (RAAS) (model 4). All covariates were selected a priori as potential confounders. The effect of SB on serum bicarbonate and creatinine clearance as well as safety endpoints (effect of SB on blood pressure, nutritional parameters, fluid retention and all-cause hospitalization) were recorded during follow-up at each study visit. Parametric (t test) and non-parametric tests (Wilcoxon sum rank test, Chi square test, Fisher exact test) were used to evaluate for between- and within-group differences according to the treatment assignment. Initial vs last available data on creatinine clearance were used to test the effect of SB on creatinine clearance. Statistical analyses were conducted as per intention to treat. For longitudinal plots on serum bicarbonate levels, SB dose and renal function complete cases are considered and no data imputation of missing data has been carried out. Furthermore, to comply with standard regulation, participants who withdrew informed consent were not analyzed. Sensitivity analysis were carried out to evaluate (1) the association of SB vs SC treatment and the occurrence of the composite endpoint of all three endpoints (creatinine doubling, all-cause mortality and dialysis inception) by applying the same approach described for survival analyses (Supplemental material A); (2) the impact on renal function decline (defined by the slope of creatinine clearance decline) of SB vs SC among patients who completed 36 months of follow-up (Supplemental material B); (3) the potential effect of any of the main demographic and clinical characteristics on the effects of sodium bicarbonate on real function or all-cause mortality (Supplemental material C). Statistical significance was defined as a p value < 0.05. Analyses were carried out with R version 3.3.2 (2016-10-31) the R Foundation for Statistical Computing for Mac.

## Results

**Fig. 1:**
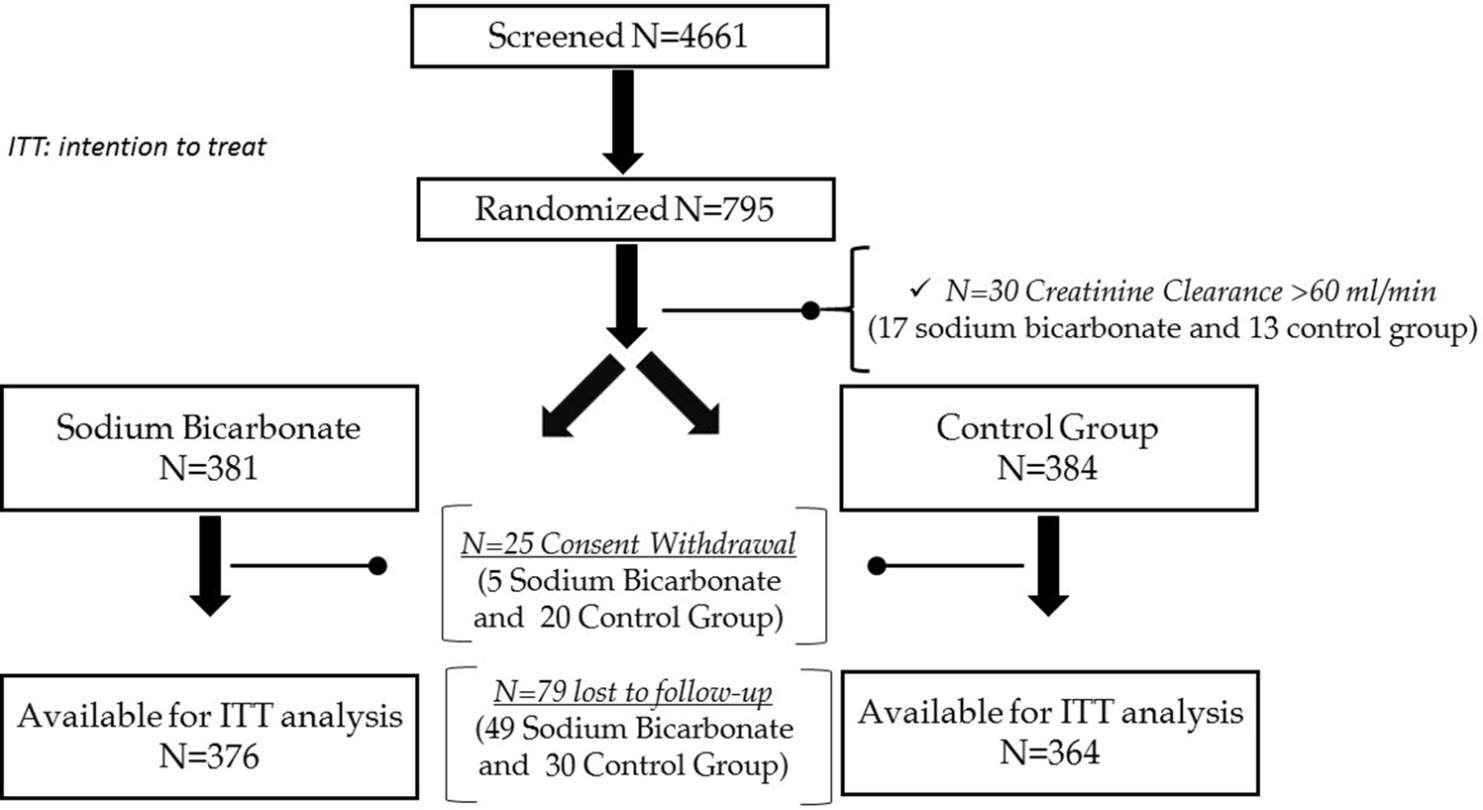
Disposition of UBI study participants

A total of 795 individuals enrolled in the UBI study (398 in SB and 397 in SC) (Fig. 1). However, 30 (13 in SC and 17 in SB) and 25 (20 in SC and 5 in SB) participants were excluded because the eGFR was > 60 ml/min/1.73 m^2^ or because they withdrew consent (according to local regulations), respectively. Of the 740 recruited individuals, 79 participants were lost to follow-up (30 in SC and 49 in SB) (Fig. 1). A final sample of 364 participants in SC and 376 participants in SB were included in intent-to-treat analyses.

### Demographic, clinical and laboratory data at baseline

Table 1 shows the baseline characteristics of the study participants. Demographic characteristics and the etiology of CKD were similar between the groups. However, those allocated to SB had lower eGFR and creatinine clearance, lower LDL and triglycerides levels, higher proteinuria, higher serum bicarbonate concentration and a higher proportion had diabetes, coronary artery disease and used calcium channel blockers (Table 1).

**Table 1:** Demographic, clinical laboratory and concomitant medication of the UBI study participants overall and according to the study drug allocation

Characteristic	Total (n = 740)	Standard Care (n = 364)	Sodium Bicarbonate (n = 376)
	Mean (SD)	Mean (SD)	Mean (SD)
Demographic characteristics			
Age (years)	67.8 (14.9)	68.1 (14.7)	67.6 (15.1)
Male sex (%)	61.8%	61.5%	62.2%
BMI (kg/m^2^)	27.9 (4.6)	28.2 (4.6)	27.7 (4.6)
Systolic blood pressure (mmHg)	128 (18)	128 (18)	129 (19)
Diastolic blood pressure (mmHg)	73 (10)	73 (8)	74 (11)
Clinical characteristics			
CKD etiology			
Unknown (%)	23.5%	24.5%	22.6%
Diabetes mellitus (%)	30.7%	27.2%	34.0%
ADPKD (%)	10.7%	11.8%	9.6%
Chronic heart failure (%)	4.9%	6.0%	3.7%
Hypertension (%)	17.7%	19.0%	16.5%
Glomerulonephropathy (%)	7.7%	6.0%	9.3%
Pyelonephritis (%)	4.9%	5.5%	4.3%
Hypertension (%)	89.2%	86.0%	92.3%
Peripheral artery disease (%)	26.5%	30.5%	22.6%
Coronary artery disease (%)	20.1%	17.3%	22.9%
Laboratory characteristics			
Hemoglobin (g/dl)	12.3 (1.7)	12.4 (1.7)	12.2 (1.8)
Serum albumin (g/dl)	3.9 (0.4)	3.9 (0.4)	3.8 (0.4)
Serum glucose (mg/dl)	119 (41)	118 (41)	120 (40)
HBa1c (%)	6.7 (1.1)	6.7 (1.1)	6.7 (1.1)
Blood urea nitrogen (mg/dl)	94 (42)	86 (36)	101 (46)
Serum creatinine (mg/dl)	2.3 (0.9)	2.1 (0.7)	2.4 (1.1)
Creatinine clearance (ml/min)	30 (12)	32 (12)	28 (11)
eGFR (ml/min/1.73 m^2^)	35.1 (11.8)	36.9 (10.8)	33.4 (12.4)
Serum bicarbonate (mmol/l)	21.5 (2.4)	21.4 (2.1)	21.7 (2.6)
Serum uric acid (mg/dl)	5.1 (1.5)	4.9 (1.3)	5.2 (1.7)
Serum potassium (mEq/l)	4.9 (0.6)	4.9 (0.6)	4.9 (0.5)
Serum sodium (mEq/l)	139 (2.9)	139 (2.8)	139 (2.9)
Cholesterol (mg/dl)	158 (36)	159 (34)	157 (37)
HDL cholesterol (mg/dl)	47 (11)	48 (11)	46 (12)
LDL cholesterol (mg/dl)	101 (29)	106 (31)	95 (25)
Triglycerides (mg/dl)	174 (85)	187 (88)	161 (80)
Serum calcium (mg/dl)	9.2 (0.6)	9.2 (0.5)	9.2 (0.7)
Serum phosphate (mg/dl)	4.0 (0.8)	3.9 (0.8)	4.0 (0.9)
Parathyroid hormone (pg/ml)	105 [68–152]	119 [66–141]	109 [70–167]
C reactive protein (mg/dl)	2.5 [1.0–3.9]	2.1 [1.1–3.5]	2.5 [1.0–4.1]
Proteinuria (mg/day)	200 [69–400]	183 [0–310]	208 [100–555]
Urinary urea (g/day)	21.0 (6.2)	22.5 (5.9)	19.5 (6.2)
Urinary phosphate (mg/day)	587 (253)	565 (234)	607 (269)
Urinary sodium (mEq/day)	165 (43)	173 (44)	157 (41)
Urinary potassium (mEq/day)	35 (19)	33 (18)	38 (20)
Concomitant medications			
Furosemide dose (mg/day)	50 [50–75]	50 [50–75]	50 [50–100]
RAAS blockade (%)			
No	10.8%	14.2%	7.4%
One drug	30.5%	29.9%	31.1%
Two drugs	58.6%	55.7%	61.4%
Calcium channel blockers (%)	30.4%	26.6%	34.0%
Number of antihypertensive drugs (N)	4 [3–5]	4 [3–5]	4 [3–5]
Paracalcitol (%)	45.8%	44.0%	47.6%
Other forms of vitamin D (%)	40.9%	41.5%	40.4%
Phosphate binders (%)	83.9%	83.2%	84.6%

Data are presented as mean (standard deviation) or median [interquartile range] as appropriate

### Adherence to study medication and achievement of serum bicarbonate target during study follow-up

The mean (SD) follow-up was 29.9 (10.3) months [29.6 (9.8) vs 30.3 (10.7) months in SC and SB, respectively]. Over the course of the study, the mean (SD) daily doses of SB was 1.13 (0.10). 1.12 (0.11) and 1.09 (0.12) mmol/kg-bw/day in the first, second and third year of follow-up, respectively. Rescue therapy with sodium bicarbonate was prescribed to 78, 26 and 33 participants in SC in the first, second and third year of follow-up, respectively. The mean (SD) dose of sodium bicarbonate prescribed as rescue therapy to those in SC was 0.28 (0.23), 0.24 (0.05) and 0.21 (0.06) mmol/kg-bw/day in the first, second and third year of follow-up, respectively. The duration participants received rescue therapy ranged from 2 to 4 months in year 1, 3–5 months in year 2, and 2–4 months in year 3.

**Fig. 2:**
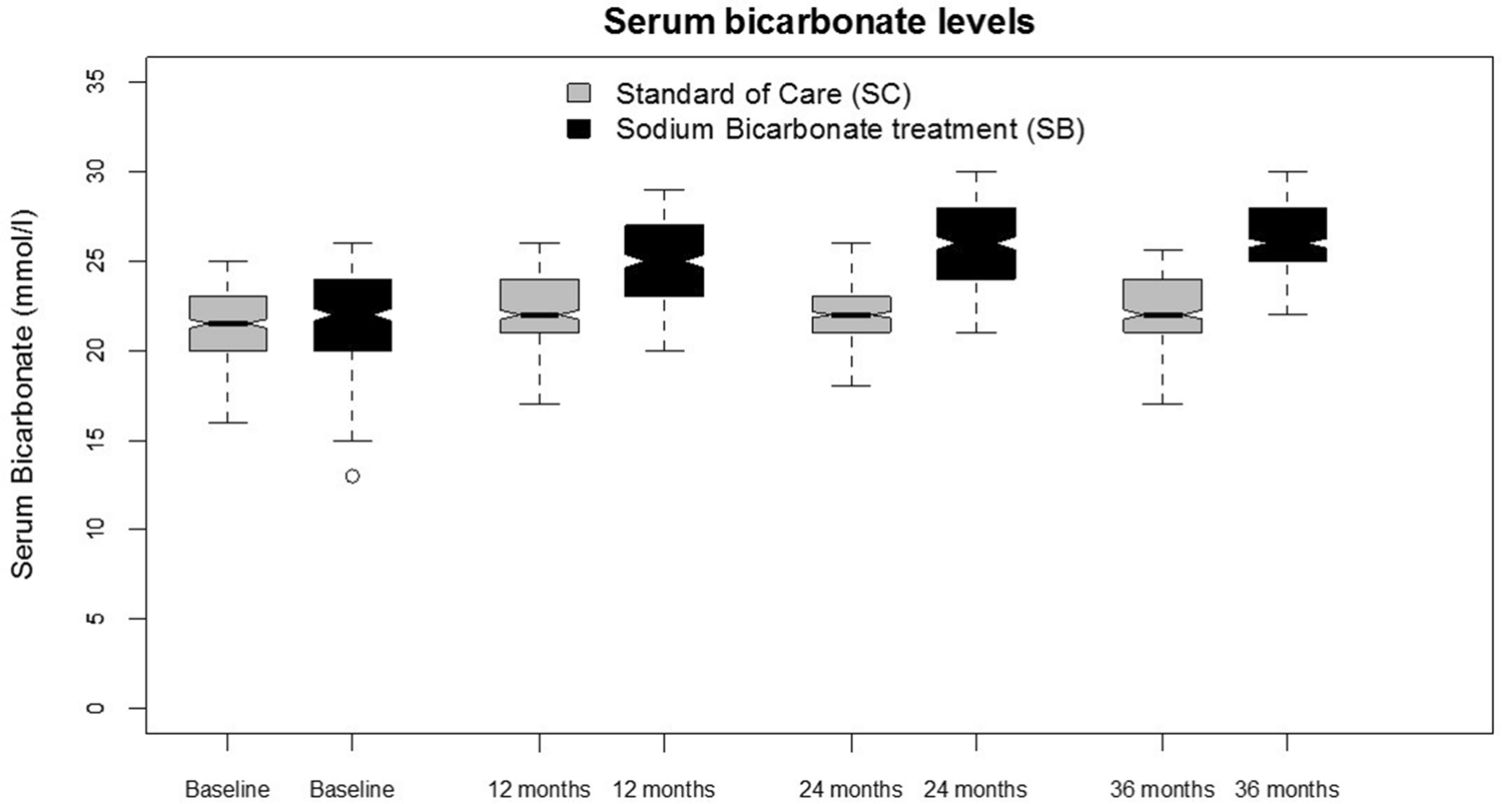
Serum levels of bicarbonate during the UBI study by treatment assignment. Analysis are carried out as per intention-to-treat and no data imputation on missing data is performed

Figure 2 shows serum bicarbonate concentration during the course of the study. At baseline, 172 (45.7%) participants in SB and 183 (50.2%) in SC group had serum levels of bicarbonate below 22 mmol/l. As depicted in Fig. 2 and Table 2, the overall serum levels of bicarbonate during follow-up were different between study groups (p < 0.001) with participants allocated to SC being maintained around 22 mmol/l and participants allocated to SB maintained around 26 mmol/l. During follow-up, the number of participants with metabolic acidosis was lower in SB (22, 10 and 0 at the end of the first, second and third year, respectively) than in SC (110, 146 and 154). As expected, none in the SC group had serum levels of bicarbonate ≥ 28 mmol/l while in the SB group this occurred in 37, 60 and 15 participants at the end of the first, second and third year of follow-up, respectively.

**Table 2:** Mean (SD) dose of sodium bicarbonate administered and mean (SD) and range (min–max) of serum bicarbonate (mmol/l) during the study according to study arm allocation

	Baseline	1^b^ year	2^b^ year	3^b^ year
Standard care (SC)				
Mean (SD) SB administered dose (mmol/kg-bw/day)	–	0.28 (0.23)^a^	0.24 (0.05)^b^	0.21 (0.06)^c^
Mean serum levels of bicarbonate (SD)(mmol/l)	21.4 (2.1)	22.3 (1.9)	21.9 (1.3)	21.9 (1.9)
Range of serum bicarbonate (min–max) (mmol/l)	16–25	17–26	18–26	17–26
Sodium bicarbonate (SB)				
Mean (SD) SB administered dose (mmol/kg-bw/day)	–	1.13 (0.10)	1.12 (0.11)	1.09 (0.12)
Mean serum levels of bicarbonate (SD)(mmol/l)	21.5 (2.4)	25.0 (2.4)	26.0 (2.4)	26.1 (1.7)
Range of serum bicarbonate (min–max) (mmol/l)	13–26	20–29	21–30	22–30
P value between group comparison	0.006	<0.001	<0.001	<0.001

^a^78 patients treated for 2 – 4 months during the first year

^b^26 patients treated for 3–5 months during second year

^c^33 patients treated for 2–4 months during third year

### Effect of sodium bicarbonate treatment on kidney end points

**Fig. 3:**
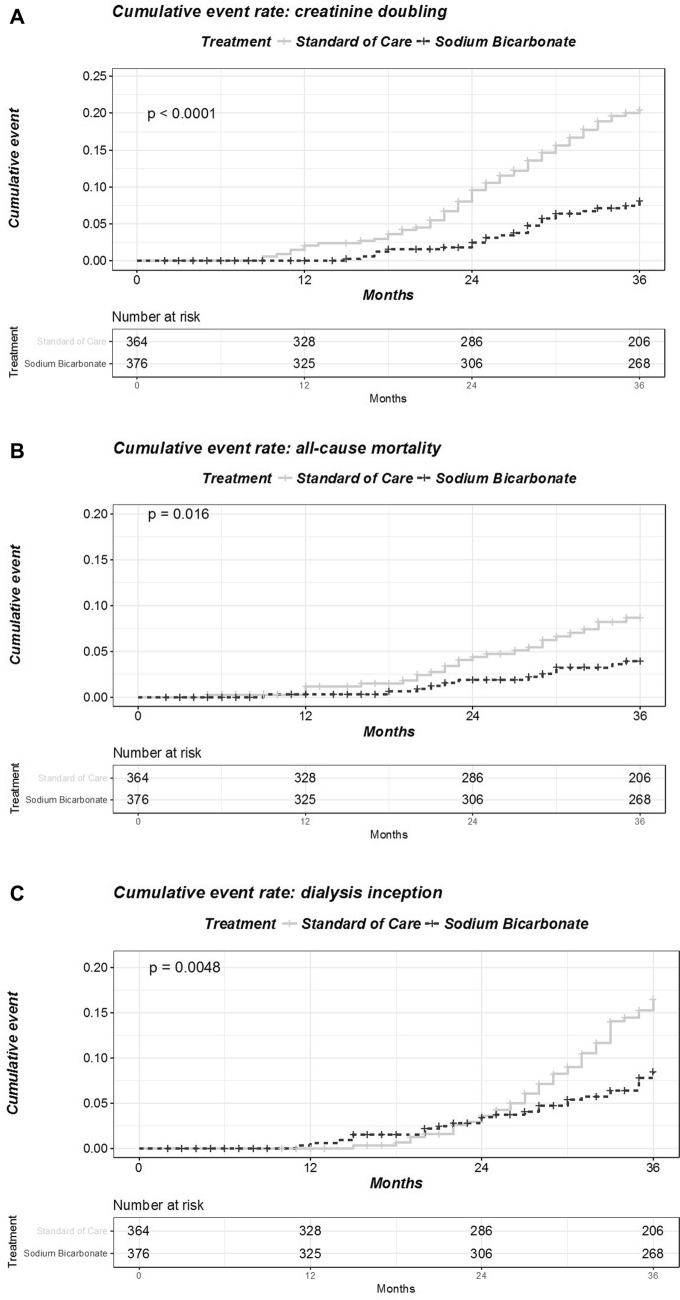
Time to **a** creatinine doubling, **b** initiation of renal replacement therapy, and **c** all-cause mortality according to the Kaplan–Meyer methods (p value refers to the unadjusted log-rank tests). Analysis are carried out as per intention-to-treat and no data imputation on missing data is performed

**Fig. 4:**
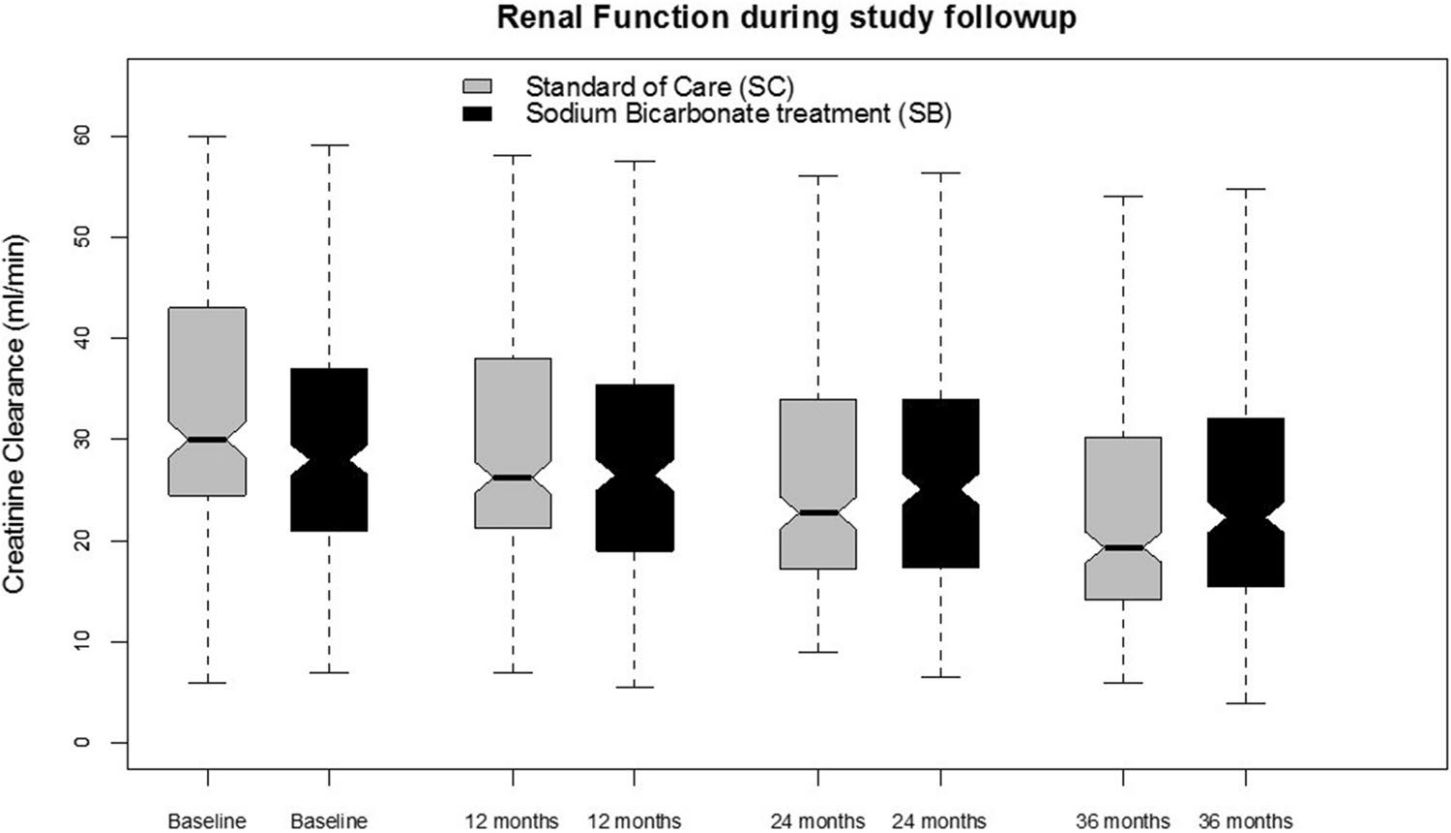
Creatinine clearance during the UBI study by treatment assignment. Analysis are carried out as per intention-to-treat and no data imputation on missing data is performed

Doubling of serum creatinine (primary endpoint) occurred in 87 participants [62 (17.0%) in SC vs 25 (6.6%) in SB, logrank test p value < 0.0001] (Fig. 3a). The Cox proportional hazard analysis showed that participants treated with SB had a 64% lower hazard rate of doubling of serum creatinine during follow-up [hazard ratio (HR) 0.36; 95% confidence interval (95% CI) 0.22–0.58; Cox-model p value < 0.001] and progressive adjustment for factors associated with the outcome of interest did not affect this association (Table 3a). At study completion, participants allocated to SC experienced a twice the decline in creatinine clearance (10.9 (5.2) vs 4.9 (4.2) in SC and SB, respectively) (Table 4, Fig. 4). Of importance, the effect of SB on creatinine clearance was maintained irrespective of baseline levels of serum bicarbonate, baseline renal function, age, gender, systolic blood pressure and proteinuria (no qualitative interaction effect Supplemental material A).

**Table 3:** Risk of creatinine doubling, dialysis initiation, and all-cause mortality with sodium bicarbonate treatment

	Hazard ratio	95% confidence interval	p value
Creatinine doubling (number of events N = 87)			
Unadjusted	0.36	0.22–0.58	< 0.001
Model 1	0.36	0.22–0.57	< 0.001
Model 2	0.36	0.22–0.58	< 0.001
Model 3	0.36	0.22–0.57	< 0.001
Model 4	0.36	0.23–0.59	< 0.001
Dialysis initiation (number of events N = 71)			
Unadjusted	0.5	0.31–0.81	0.005
Model 1	0.5	0.31–0.81	0.005
Model 2	0.49	0.30–0.80	0.004
Model 3	0.34	0.20–0.56	< 0.001
Model 4	0.28	0.16–0.48	< 0.001
All-cause mortality (number of events N = 37)			
Unadjusted	0.43	0.22–0.87	0.01
Model 1	0.43	0.21–0.86	0.018
Model 2	0.42	0.21–0.85	0.015
Model 3	0.38	0.18–0.77	0.007
Model 4	0.36	0.18–0.74	0.005

Model 1 adjusted for age and sex; model 2 adjusted for model 1 and demographics, body mass index, and blood pressure. Model 3: adjusted for model 2 and comorbidities (renal function. cardiovascular disease, peripheral arterial disease, diabetes, hypertension). Model 4: adjusted for model 3 and proteinuria, use of renin–angiotensin–aldosterone system inhibitors

**Table 4:** Change in creatinine clearance during the study by CKD stage and treatment assignment

Creatinine clearance	Baseline	12 months	24 months	Study completion	Delta from baseline		p value*	p value^^^
					ml/min	%		
Overall								
Standard of care (SC)	32.3 (12.1)	28.9 (11.9)	25.1 (11.6)	22.6 (11.2)	10.9 (5.2)	34 (16)	< 0.001	0.0287
Sodium bicarbonate (SB)	28.3 (11.9)	27.6 (11.1)	26.2 (11.1)	24.6 (10.9)	4.9 (4.2)	17 (15)	< 0.001	
CKD-3a (n = 88)								
Standard of care (SC)	42.1 (12.2)	38.0 (12.2)	33.7 (11.8)	31.1 (11.3)	11.9 (5.6)	29 (13)	< 0.001	0.572
Sodium bicarbonate (SB)	35.7 (11.2)	33.8 (11.4)	31.8 (11.4)	29.6 (12.5)	5.4 (3.7)	18 (15)	< 0.001	
CKD-3b (n = 319)								
Standard of care (SC)	34.1 (12.1)	30.5 (12.1)	26.6 (11.8)	24.5 (11.5)	10.9 (5.1)	32 (16)	< 0.001	0.002
Sodium bicarbonate (SB)	34.1 (10.8)	31.8 (10.6)	30.1 (11.0)	28.7 (10.8)	5.1 (2.9)	17 (10)	< 0.001	
CKD-4 (n = 274)								
Standard of care (SC)	27.8 (8.4)	24.5 (8.2)	20.7 (8.0)	17.5 (7.9)	10.8 (5.0)	39 (15)	< 0.001	0.006
Sodium bicarbonate (SB)	25.1 (9.0)	23.6 (8.3)	22.0 (8.2)	20.4 (7.9)	4.8 (5.2)	18 (15)	< 0.001	
CKD-5 (n = 58)								
Standard of care (SC)	14.9 (5.5)	12.9 (5.0)	9.9 (3.4)	7.7 (1.1)	7.5 (6.2)	44 (16)	< 0.001	0.001
Sodium bicarbonate (SB)	13.5 (6.2)	13.6 (6.0)	13.5 (6.0)	12.8 (5.5)	3.6 (5.9)	14 (29)	< 0.001	

The mean value of creatinine clearance (standard deviation) at each time point and the mean change (expressed as ml/min or % change) from baseline and study completion is reported. Within group comparison is made between creatinine clearance at baseline and study completion. Between groups comparison is made at study completion. Analysis are based on complete cases

*p value for comparison between baseline and completion (t test), ^^^p value for comparison between treatment groups at study completion (t test)

Time to initiation of renal replacement therapy (RRT) was also significantly different between groups. At the end of the study, 71 participants [45 (12.3%) in SC and 26 (6.9%) in SB, Log-rank test p value = 0.004] initiated RRT (Fig. 3b). The Cox proportional hazard analysis showed that participants treated with SB had a 50% lower HR of initiating dialysis during follow-up [HR 0.5; 95% CI 0.31–0.81; Cox-model p value = 0.005] and progressive adjustment for factors associated with the outcome of interest did not affect this association (Table 3b). Similarly to what reported for the primary endpoint, no qualitative interaction between baseline variables and the effect of SB treatment was noted (Supplemental material A).

The impact of SB vs SC on renal function was also assessed in patients who completed 36 months of follow-up and did not experience any of the study endpoints. Treatment with SB was associated with a significantly lower CKD progression (− 1.4 vs − 3.4 ml/min/year creatinine clearance decline in SB vs SC, respectively) irrespective of the multiple adjustment for confounders, further corroborating the results of the UBI study primary endpoint (Supplemental material B).

### Effect of sodium bicarbonate on mortality and hospitalizations

Thirty-seven participants died during follow-up; 25 (6.8%) in SC and 12 (3.1%) in SB (log-rank test p value = 0.016) (Fig. 3c). The survival analysis showed that participants treated with SB had a 57% lower HR of all-cause mortality during follow-up [HR 0.43; 95% CI 0.22–0.87; Cox-model p value = 0.01] and progressive adjustment for factors associated with the outcome of interest did not affect this association (Table 3c). No qualitative interaction between baseline variables and the effect of SB treatment was noted (Supplemental material A).

To corroborate the efficacy of SB over SC and exclude the possibility that current analysis are biased by correlated events censoring and different creatinine testing between study arms, we also explored to impact of SB on the composite endpoint of creatinine doubling, all-cause mortality and dialysis inception. As reported in the Supplemental material C, subjects allocated to SC experienced three time the risk of the occurrence of the composite endpoint as compared to subjects allocated to SB (HR 0.35; 0.25–0.48; p < 0.0001).

Similarly, rates of hospitalizations and length of stay progressively decreased among participants treated with SB while no difference was noted among patients allocated to SC (Table 5). At study completion, lower proportion in SB were hospitalized (34.6% vs 14.2%, p for between group comparison < 0.001 in SC and SB, respectively) and the duration of hospitalization was lower in SB as well (1160 vs 400 day/year, p for between group comparison < 0.001 in SC and SB, respectively).

**Table 5:** Hospitalization rate and length of hospital stay by treatment assignment during followup

	First year	Second year	Third year
Standard care			
Number hospitalized	92	81	84
Proportion hospitalized (%)	26.6	33.6	34.6
Number of days in the hospital	1085	975	1160
Sodium bicarbonate			
Number hospitalized	101	38	35
Proportion hospitalized (%)	29.7	15.4	14.2
Number of days in the hospital	1168	422	400

### Effect of sodium bicarbonate on safety endpoint

The effect of SB on safety endpoints is summarized in Table 6. In particular, no significant effect of SB on systolic or diastolic BP or total body weight was observed. No signs of fluid overload were detected and body weight was not different between the two groups during follow-up (Table 6). Overall, systolic BP decreased by 1.5 (19.8) mmHg in SB and by 1.2 (10.9) mmHg in SC. Diastolic BP decreased by 3.0 (11.8) mmHg in SB and by 2.5 (10.1) mmHg in SC (Table 6). At the end of the 36 months of follow-up, 15.6 and 11.0% in SC and SB, respectively, had systolic BP > 145 mmHg; whereas diastolic BP was > 85 mmHg in 2.4% and 7.6% in SC and SB, respectively). At baseline the mean number of antihypertensive medications as well as dose of diuretics prescribed was greater among participants allocated to SB (Table 1). During follow-up, 81 vs 68 participants increased anti-hypertensive drugs in SC and SB, while 91 and 122 decreased the number of medications to control hypertension in SC and SB groups, respectively.

**Table 6:** Safety endpoints according to study allocation

	Sodium bicarbonate			Standard care	
	Baseline	Follow-up	p value	Baseline	Follow up
Mean systolic blood pressure mmHg (SD)	129 (19)	127 (16)	0.147	128 (18)	127 (15)
Mean systolic blood pressure change mmHg	− 1.5 (19.8)			− 1.2 (10.9)	
Mean diastolic blood pressure mmHg (SD)	73 (8)	74 (9)	0.202	74 (11)	76 (10)
Mean diastolic blood pressure change mmHg	− 3.0 (11.0)			− 2.5 (10.1)	
Prevalence of systolic blood pressure N (%)					
> 145 mmHg	53 (23.3)	36 (11.0)	0.001	86 (25.7)	52 (15.6)
135–145 mmHg	51 (15.6)	49 (15.0)	0.916	34 (10.2)	61 (18.3)
< 100 mmHg	32 (9.8)	19 (5.8)	0.077	17 (5.1)	11 (3.3)
Prevalence of diastolic blood pressure N (%)					
> 85 mmHg	28 (8.6)	25 (7.6)	0.743	10 (3.0)	8 (2.4)
Serum albumin (g/dl)	3.8 (0.4)	3.9 (0.4)	0.005	3.8 (0.4)	3.8 (0.5)
Serum hemoglobin (g/dl)	12.2 (1.8)	12.4 (2.1)	0.499	12.4 (1.7)	12.3 (1.8)
Serum potassium (mmol/l)	4.9 (0.5)	4.9 (0.6)	0.518	4.9 (0.6)	4.9 (0.7)
24 h urine sodium (mmol/l)	157 (41)	154 (23)	0.384	173 (44)	159 (41)
24 h chloride (mmol/l)	128 (22)	98 (21)	0.001	124 (20)	119 (24)
Body weight (kg)	74.7 (13.0)	74.0 (11.7)	0.467	75.6 (9.7)	74.9 (11.7)

The mean (standard deviation) of baseline as well as the last available data at follow-up is reported. Complete cases are reported and no missing data imputation is performed

No major between group differences in electrolytes were apparent with the exception of urinary sodium and chloride excretion (Table 6). When compared to SC, participants allocated to SB had lower urinary sodium excretion at baseline. However, no between groups difference was noted at study completion (Table 6). Indeed, sodium intake was similar in the 2 study arms during follow-up (9.0 vs 8.8 g/day in SB and SC group. respectively) and urinary chloride excretion was markedly lower in SB than in SC during follow-up, suggesting that participants in SB reduced sodium chloride intake. Notably, no signs of hemodilution were apparent. At baseline, no meaningful difference in serum levels of albumin were observed between the groups (Table 1). During follow-up, only 78 participants (38 in SC and 40 in SB) showed albumin levels lower than 3.5 g/dl during follow-up. Similarly, serum hemoglobin was 12.4 (1.7) g/dl and 12.2 (1.8) g/dl in the SC and in the SB group at baseline (Table 1). Ten participants in SC and 11 in SB experienced a reduction in hemoglobin levels below 9.5 g/dl while 109 participants in SC and 90 in SB had hemoglobin levels higher than 13 g/dl. A total of 403 participants (205 in SC and 198 in SB) received erythropoietin during study follow-up.

## Discussion

Metabolic acidosis is a complication of CKD associated with increased protein catabolism [4, 13], branched-chain amino-acids oxidation [14], reduced insulin sensitivity [3, 4, 15], and bone demineralization [16, 17]. More recent evidence has linked metabolic acidosis with CKD progression [18, 19], poor cognitive function and poor kidney allograft survival [20]. Although experimental models have shown beneficial effects of correcting metabolic acidosis with alkali supplementation on the kidney [21–23], evidence from well-designed, large-scale clinical trials is lacking. Hence, the Use of Bicarbonate in Chronic Renal Insufficiency (UBI) study was conducted to test the hypothesis that correction of metabolic acidosis with sodium bicarbonate preserves kidney function and reduces mortality in CKD. The main results of the UBI study confirm that treatment of metabolic acidosis with sodium bicarbonate in patients with CKD stage 3–5 is safe and improves kidney and patient survival. Indeed, untreated participants had a threefold risk of creatinine doubling and a twofold higher risk of starting dialysis, respectively. Correction of CKD-related metabolic acidosis preserved kidney function irrespective of gender and several other notable characteristics including the use of RAAS inhibitors.

The UBI findings confirm those of other studies with smaller sample size. In a single center, open-label randomized, controlled trial of 134 individuals with CKD, treatment of metabolic acidosis with sodium bicarbonate delayed progression of CKD and improved nutritional status [24]. Phistkul et al., also reported that correction of metabolic acidosis preserved the eGFR in a smaller study of 29 individuals treated with sodium citrate compared to 30 individuals unable or unwilling to take the treatment over 24 months of follow-up [25]. Thus, the available interventional evidence supports the hypothesis that treatment of metabolic acidosis preserves kidney function in CKD.

There is also intriguing evidence suggesting that oral bicarbonate supplementation may preserve kidney function in CKD patients with normal serum bicarbonate concentration. Mahajan et al. reported that sodium bicarbonate supplementation significantly preserved kidney function in 120 individuals with early hypertensive nephropathy and normal serum bicarbonate concentration [26]. Large-scale clinical trials to determine whether alkali supplementation preserves kidney function in CKD patients with normal serum bicarbonate are warranted, particularly since the vast majority of CKD patients have normal serum bicarbonate concentration [1] and are not treated with oral alkali based on current practice standards.

Several mechanisms have been identified that link metabolic acidosis with CKD progression. Experimental data suggest that metabolic acidosis triggers endothelin production and activation of its receptors type A and B as well as promotes the complement cascade activation [27, 28]. Other lines of evidence suggest that acidosis stimulates angiotensin II production and upregulates TGF-beta further linking renal function loss with acidosis [21–23]. Hence, impaired renal acid excretion and excessive dietary acid intake contribute to metabolic acidosis and progressive renal function loss. In animal models, the use of fruit and vegetables as well as sodium citrate supplementation have been demonstrated to prevent the occurrence of metabolic acidosis and attenuate endothelin- and aldosterone-induced fibrosis and delay residual renal function loss [29–31]. We also demonstrate for the first time that correction of metabolic acidosis reduces risk of all-cause mortality in CKD (fully adjusted HR 0.36; 95% CI 0.18–0.74). Although the mechanisms are uncertain, this may be in part due to the positive impact of alkali supplementation on renal function. Results from observational studies have found that serum bicarbonate levels ≥ 27 mmol/l are associated with an increased risk of incident heart failure [32], raising the concern that increasing serum bicarbonate levels towards this range might be associated with adverse cardiovascular consequences. Nonetheless, in the UBI study, efforts to achieve a serum bicarbonate levels of 24–28 mmol/l improved survival and no significant differences in blood pressure, total body weight or hospitalizations were noted. In spite of the administration of a considerable dose of sodium bicarbonate (0.8–1.1 mmol/kg/day—about 1.8 g/day of elemental sodium), we did not observe a significant increase in BP. However, it should be mentioned that the overall sodium intake in the two study arms was similar (about 9 g/day). This suggests that those in SB reduced their sodium chloride intake in response to the nutritional counselling performed as part of the UBI study protocol. Nevertheless, we did not observed significant differences in BP during this clinical trial, which is consistent with previous experiences [24, 26], and supports the hypothesis that sodium-mediated fluid retention occurs when sodium is accompanied by the chloride anion and not the bicarbonate anion [33, 34].

The UBI study is the largest, randomized, pragmatic clinical trial evaluating the effect of sodium bicarbonate supplementation in patients with CKD. Whereas prior published trials were single-center in nature that mostly enrolled patients with hypertensive CKD, the UBI study was a multicenter trial that enrolled patients with diverse etiologies of CKD. Participants were also followed for a mean (SD) duration of 29.9 (10.3) months. Nevertheless, the study has some limitations. The limited budget of the UBI study, did not allow for implementing the use of placebo or adoption of common protocols for patients care among centers (including the decision to start dialysis) and participants as well as physicians were aware of the treatment administered. Accordingly, the follow-up schedule was decided by the attending physician according to the standard practice in use at the study center. However, while these may have resulted in censoring or treatment bias, the sensitivity analysis (i.e. incidence of the composite endpoint as well as creatinine slope decline among patients followed for 36 months) corroborate the main findings of the UBI study. Although diet may influence sodium bicarbonate levels [35, 36], no specific nutritional regimen was enforced in the UBI study. However, alkali therapy was titrated to achieve a serum bicarbonate level between 24 and 28 mmol/l, minimizing the potential interaction of nutrition on serum levels of bicarbonate. Furthermore, the random assignment of the alkali treatment in the UBI study and the progressive adjustment of analyses for measured factors should reassure against any potential confounding effect of nutrition on current results. Similarly, drug or diet adherence was not monitored. Rescue therapy with sodium bicarbonate occurred in a large proportion of participants in the control group. Nevertheless, the duration of treatment was short for those who received rescue treatment and this would have biased the results towards the null. There were relatively few mortality events, so the effect of treatment of metabolic acidosis with sodium bicarbonate on mortality should be interpreted with this in mind. Gastrointestinal intolerance was not investigated and hence the UBI study cannot shed light on this aspect. However, the relatively low (14.1%). equally balanced between groups as well as expected dropout rate should reassure on the overall safety of SB even at the dosages used in the UBI study (mean daily dose of sodium bicarbonate and elemental sodium: ~ 6.8 g/day and ~ 1.8 g/day, respectively). Pill burden may be another factor that could limit implementation of current results. Finally, the relatively selected population studied and the fact that this study was conducted in Italy should caution against the generalizability of current findings and whether the UBI study results apply to other populations with different dietary habits and standard of care is uncertain.

In conclusion, correction of metabolic acidosis with oral sodium bicarbonate is safe and reduces the risk of CKD progression and all-cause mortality in patients with CKD 3–5 without advanced stages of chronic heart failure.

## Electronic supplementary material

Below is the link to the electronic supplementary material.

Supplementary material 1 (PDF 541 kb)

## References

[1] Moranne O, Froissart M, Rossert J (2009). Timing of onset of CKD-related metabolic complications. J Am Soc Nephrol.

[2] Kidney Disease: Improving Global Outcomes (KDIGO) CKD Work Group (2012). KDIGO clinical practice guideline for the evaluation and management of chronic kidney disease. Kidney Int Suppl.

[3] Bellasi A, Di Micco L, Santoro D (2016). Correction of metabolic acidosis improves insulin resistance in chronic kidney disease. BMC Nephrol.

[4] Garibotto G, Sofia A, Russo R (2015). Insulin sensitivity of muscle protein metabolism is altered in patients with chronic kidney disease and metabolic acidosis. Kidney Int.

[5] Roderick P, Willis NS, Blakeley S (2007). Correction of chronic metabolic acidosis for chronic kidney disease patients. Cochrane Database Syst Rev.

[6] Navaneethan SD, Shao J, Buysse J (2019). Effects of treatment of metabolic acidosis in CKD: a systematic review and meta-analysis. Clin J Am Soc Nephrol.

[7] Aigner C, Cejka D, Sliber C (2019). Oral sodium bicarbonate supplementation does not affect serum calcification propensity in patients with chronic kidney disease and chronic metabolic acidosis. Kidney Blood Press Res.

[8] Tanemoto M (2019) Gap acidosis except lactic acidosis develops and progresses during kidney disease stage G5. Clin Exp Nephrol 1045–1049 10.1007/s10157-019-01743-4 31062197

[9] Goraya N, Munoz-Maldonado Y, Simoni J (2019). Fruit and vegetable treatment of chronic kidney disease-related metabolic acidosis reduces cardiovascular risk better than sodium bicarbonate. Am J Nephrol.

[10] Di Iorio B, Aucella F, Conte G (2012). A prospective, multicenter, randomized, controlled study: the correction of metabolic acidosis with use of bicarbonate in chronic renal insufficiency (UBI) Study. J Nephrol.

[11] Levey AS, Stevens LA, Schmid CH (2009). A new equation to estimate glomerular filtration rate. Ann Intern Med.

[12] Maroni BJ, Steinman TI, Mitch WE (1985). A method for estimating nitrogen intake of patients with chronic renal failure. Kidney Int.

[13] Mitch WE, Du J, Bailey JL (1999). Mechanisms causing muscle proteolysis in uremia: the influence of insulin and cytokines. Miner Electrolyte Metab.

[14] Lofberg E, Wernerman J, Anderstam B (1997). Correction of acidosis in dialysis patients increases branched-chain and total essential amino acid levels in muscle. Clin Nephrol.

[15] Kobayashi S, Maesato K, Moriya H (2005). Insulin resistance in patients with chronic kidney disease. Am J Kidney Dis.

[16] Bushinsky DA, Chabala JM, Gavrilov KL (1999). Effects of in vivo metabolic acidosis on midcortical bone ion composition. Am J Physiol.

[17] Krieger NS, Sessler NE, Bushinsky DA (1992). Acidosis inhibits osteoblastic and stimulates osteoclastic activity in vitro. Am J Physiol.

[18] Tammaro G, Zacchia M, Zona E (2018). Acute and chronic effects of metabolic acidosis on renal function and structure. J Nephrol.

[19] Di Iorio BR, Cupisti A, D’Alessandro C (2018). Nutritional therapy in autosomal dominant polycystic kidney disease. J Nephrol.

[20] Park S, Kang E, Park S (2017). Metabolic acidosis and long-term clinical outcomes in kidney transplant recipients. J Am Soc Nephrol.

[21] Wesson DE, Nathan T, Rose T (2007). Dietary protein induces endothelin-mediated kidney injury through enhanced intrinsic acid production. Kidney Int.

[22] Wesson DE, Simoni J, Prabhakar S (2006). Endothelin-induced increased nitric oxide mediates augmented distal nephron acidification as a result of dietary protein. J Am Soc Nephrol.

[23] Wesson DE, Jo CH, Simoni J (2015). Angiotensin II-mediated GFR decline in subtotal nephrectomy is due to acid retention associated with reduced GFR. Nephrol Dial Transplant.

[24] de Brito-Ashurst I, Varagunam M, Raftery MJ (2009). Bicarbonate supplementation slows progression of CKD and improves nutritional status. J Am Soc Nephrol.

[25] Phisitkul S, Khanna A, Simoni J (2010). Amelioration of metabolic acidosis in patients with low GFR reduced kidney endothelin production and kidney injury, and better preserved GFR. Kidney Int.

[26] Mahajan A, Simoni J, Sheather SJ (2010). Daily oral sodium bicarbonate preserves glomerular filtration rate by slowing its decline in early hypertensive nephropathy. Kidney Int.

[27] Nath KA, Hostetter MK, Hostetter TH (1985). Pathophysiology of chronic tubulo-interstitial disease in rats. Interactions of dietary acid load, ammonia, and complement component C3. J Clin Investig.

[28] Phisitkul S, Hacker C, Simoni J (2008). Dietary protein causes a decline in the glomerular filtration rate of the remnant kidney mediated by metabolic acidosis and endothelin receptors. Kidney Int.

[29] Goraya N, Wesson DE (2015). Dietary interventions to improve outcomes in chronic kidney disease. Curr Opin Nephrol Hypertens.

[30] Goraya N, Simoni J, Jo CH (2014). Treatment of metabolic acidosis in patients with stage 3 chronic kidney disease with fruits and vegetables or oral bicarbonate reduces urine angiotensinogen and preserves glomerular filtration rate. Kidney Int.

[31] Wesson DE, Simoni J (2010). Acid retention during kidney failure induces endothelin and aldosterone production which lead to progressive GFR decline, a situation ameliorated by alkali diet. Kidney Int.

[32] Dobre M, Yang W, Chen J (2013). Association of serum bicarbonate with risk of renal and cardiovascular outcomes in CKD: a report from the chronic renal insufficiency cohort (CRIC) study. Am J Kidney Dis.

[33] Schmidlin O, Tanaka M, Sebastian A (2010). Selective chloride loading is pressor in the stroke-prone spontaneously hypertensive rat despite hydrochlorothiazide-induced natriuresis. J Hypertens.

[34] Kotchen TA, Kotchen JM (1997). Dietary sodium and blood pressure: interactions with other nutrients. Am J Clin Nutr.

[35] Cupisti A, Brunori G, Di Iorio BR (2018). Nutritional treatment of advanced CKD: twenty consensus statements. J Nephrol.

[36] Bellizzi V, Conte G, Borrelli S (2017). Controversial issues in CKD clinical practice: position statement of the CKD-treatment working group of the Italian Society of Nephrology. J Nephrol.

