# Correction to: Treatment of metabolic acidosis with sodium bicarbonate delays progression of chronic kidney disease: the UBI Study

**DOI:** 10.1007/s40620-020-00742-z

**Published:** 2020-05-07

**Authors:** Biagio R. Di Iorio, Antonio Bellasi, Kalani L. Raphael, Domenico Santoro, Filippo Aucella, Luciano Garofano, Michele Ceccarelli, Luca Di Lullo, Giovanna Capolongo, Mattia Di Iorio, Pasquale Guastaferro, Giovambattista Capasso, The UBI Study Group, Vincenzo Barbera, Annamaria Bruzzese, Valeria Canale, Giuseppe Conte, Vincenzo Crozza, Adamasco Cupisti, Antonella De Blasio, Emanuele De Simone, Lucia Di Micco, Fulvio Fiorini, Rachele Grifa, Raffaella Nardone, Matteo Piemontese, Maria Luisa Sirico, Fabio Vitale

**Affiliations:** 1 Nephrology and Dialysis Unit, PO “A. Landolfi”, Solofra, Avellino Italy; 2 Department of Research, Innovation, Brand Reputation, Bergamo Hospital, ASST Papa Giovanni XXIII, Bergamo, Italy; 3 Division of Nephrology and Hypertension, Department of Internal Medicine, University of Utah Health, Salt Lake City, UT USA; 4 Dialysis and Nephrology Unit, University of Messina, Messina, Italy; 5 Department of Nephrology and Dialysis, IRCCS “Casa Sollievo della Sofferenza”, San Giovanni Rotondo, Foggia, Italy; 6 Biogem, Section of Genetic and Translational Medicine, Ariano Irpino, Avellino Italy; 7 Department of Science and Technology, University of Sannio, Benevento, Italy; 8 Department of Nephrology and Dialysis, “L. Parodi-Delfino” Hospital, Colleferro, Roma, Italy; 9 Department of Translational Medical Sciences, University of Campania “Luigi Vanvitelli”, Naples, Italy; 10 Data Scientist, Landolfi Nephrology Dialysis Consultant, Solofra, Avellino, Italy; 11 Department of Nephrology, “G. Criscuoli” Hospital, Sant’Angelo dei Lombardi, Avellino Italia; 12 Nephrology and Dialysis, “Antonio Cardarelli” hospital, Naples, Italia

## Correction to: Journal of Nephrology (2019) 32:989–1001 10.1007/s40620-019-00656-5

It occurred to us that a simple but significant calculation error was made in Table 2 in the dose of bicarbonate administered. Indeed, contrary to what reported in Table 2, the dose of sodium bicarbonate administered during study was:

Mean (SD) SB administered dose (mmol/kg bw/day)

**Table:** 

	Baseline	1 year	2 year	3 year
SC	–	0.14 (0.12)	0.12 (0.03)	0.11 (0.03)
SB	–	0.56 (0.05)	0.55 (0.05)	0.54 (0.06)

We sincerely apologize for the inconvenience. The updated Table 2 has been copied below:

**Table 2:** Mean (SD) dose of sodium bicarbonate administered and mean (SD) and range (min–max) of serum bicarbonate (mmol/l) during the study according to study arm allocation

	Baseline	1^b^ year	2^b^ year	3^b^ year
Standard care (SC)	–	0.14 (0.12)	0.12 (0.03)	0.11 (0.03)
Mean (SD) SB administered dose (mmol/kg-bw/day)	–	0.28 (0.23)^a^	0.24 (0.05)^b^	0.21 (0.06)^c^
Mean serum levels of bicarbonate (SD) (mmol/l)	21.4 (2.1)	22.3 (1.9)	21.9 (1.3)	21.9 (1.9)
Range of serum bicarbonate (min–max) (mmol/l)	16–25	17–26	18–26	17–26
Sodium bicarbonate (SB)	–	0.56 (0.05)	0.55 (0.05)	0.54 (0.06)
Mean (SD) SB administered dose (mmol/kg-bw/day)	–	1.13 (0.10)	1.12 (0.11)	1.09 (0.12)
Mean serum levels of bicarbonate (SD) (mmol/l)	21.5 (2.4)	25.0 (2.4)	26.0 (2.4)	26.1 (1.7)
Range of serum bicarbonate (min–max) (mmol/l)	13–26	20–29	21–30	22–30
P value between group comparison	0.006	<0.001	<0.001	<0.001

^a^78 patients treated for 2 – 4 months during the first year

^b^26 patients treated for 3–5 months during second year

^c^33 patients treated for 2–4 months during third year

