# Supplementary material for: Treatment of metabolic acidosis with sodium bicarbonate delays progression of chronic kidney disease: the UBI Study
Source: J Nephrol. 2019 Oct 9;32(6):989–1001. doi: 10.1007/s40620-019-00656-5 (PMC6821658; doi:10.1007/s40620-019-00656-5)
Supplement: Supplementary file 1 — Supplementary material 1 (PDF 541 kb) [file 40620_2019_656_MOESM1_ESM.pdf]

Supplemental material

Table of contents:

(A) Effects of sodium bicarbonate (SB) treatment on the composite endpoint of creatinine doubling, dialysis inception and all-cause mortality.

(B) Effects of sodium bicarbonate (SB) treatment on renal function decline defined by the slope of creatinine clearance decline

(C) Supplementary analysis to explore the potential effect of any of the main demographic and clinical characteristics on the effects of sodium bicarbonate on real function or all-cause mortality

---

(A) Effects of sodium bicarbonate (SB) treatment on the composite endpoint of creatinine doubling, dialysis inception and all-cause mortality.

The cumulative incidence curves were constructed according to the Kaplan-Meier method and the log rank test was used to determine statistical significance.

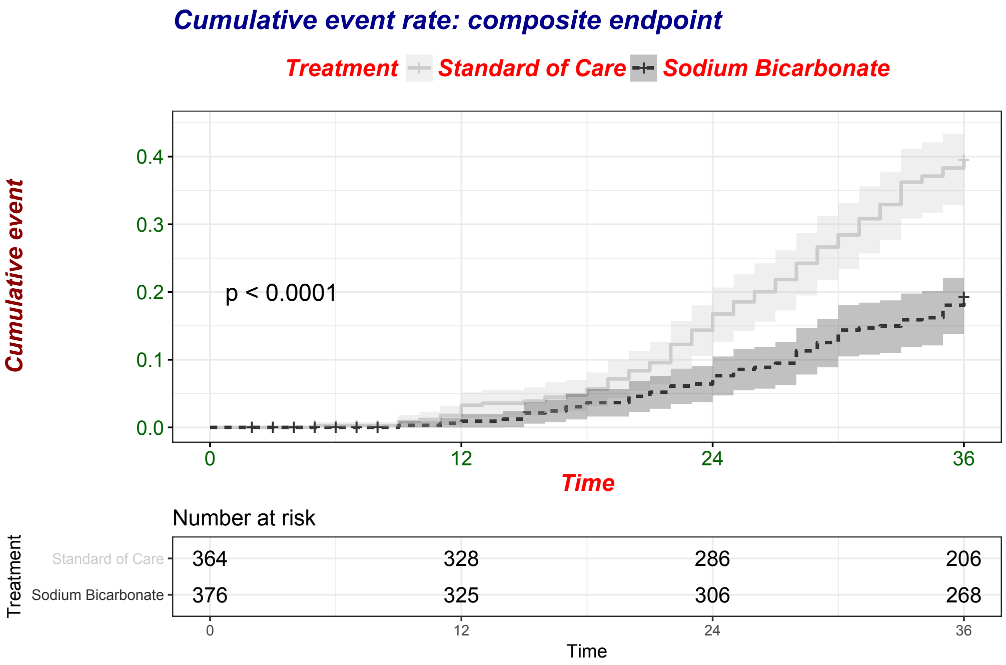

To explore the association of sodium bicarbonate treatment and the time to the composite endpoint time, Cox proportional-hazard model was utilized. Cox proportional hazard regression analysis are presented as: (i) unadjusted; (ii) adjusted for age and sex (model 1); (iii) adjusted for model 1 and body mass index (BMI), systolic and diastolic BP (model 2); (iv) adjusted for model 2 and baseline creatinine clearance, cardiovascular disease (CVD), diabetes, hypertension (model 3); (v) adjusted for model 3 and proteinuria and use of medications that inhibit the renin-angiotensin-aldosterone system (RAAS) (model 4). All covariates were selected *a priori* as potential confounders.

	Hazard Ratio (95% Confidence Interval)	p-value
Unadjusted	0.425 (0.315 – 0.575)	<.0001
Model 1	0.425 (0.315 – 0.574)	<.0001
Model 2	0.423 (0.313 – 0.571)	<.0001
Model 3	0.366 (0.269 – 0.498)	<.0001
Model 4	0.353 (0.259 – 0.481)	<.0001

The Cox proportional hazard assumption was verified in the unadjusted model by by using the Schoenfeld residuals against the transformed time (rho: -0.00781; chisq 0.0119; p= 0.913). Statistical significance was defined as a p-value <0.05. Analyses were carried out with R version 3.3.2 (2016-10-31), the R Foundation for Statistical Computing for Mac.

(B) Effects of sodium bicarbonate (SB) treatment on renal function decline defined by the slope of creatinine clearance decline

In the UBI study renal function was assessed via the 24-hours creatinine clearance. To further elucidate the effect of sodium bicarbonate (SB) treatment on renal function, we calculate the slope of the annual decline of creatinine clearance among patients that completed 36 moths of follow-up. For current analyses we considered data of complete cases and no missing data imputation has been carried out.

Renal function decline (slope – ml/min/year)	Median	Interquartile range
Overall	-1.970	-3.520; -1.380
Allocated to sodium bicarbonate	-1.420	-1.645; -1.215
Allocated to standard of care	-3.475	-4.248; -2.722

Patients treated with SB had a lower annual decline in renal function as also illustrated in the boxplot below.

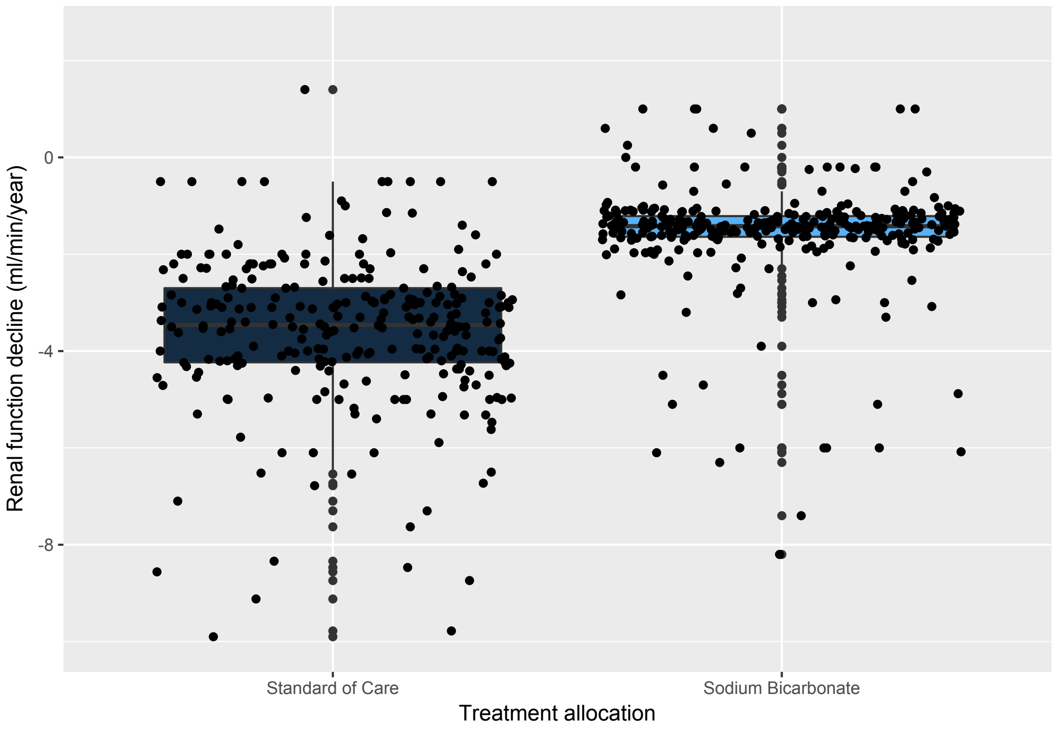

To better explore the effect of SB supplementation (independent variable) on renal function decline (dependent variable), linear regression models were utilized. The regression models were progressively adjusted for potential confounders:

	Beta coefficient	Std.Error	t value	P-value
Unadjusted	2.029	0.131	15.46	<2e-16 ***
Model 1	2.029	0.131	15.46	<2e-16 ***
Model 2	1.997	0.131	15.246	< 2e-16 ***
Model 3	1.844	0.127	14.487	< 2e-16 ***
Model 4	1.856	0.126	14.616	< 2e-16 ***

Model 1 adjusted for age and sex;

Model 2 adjusted for model 1 and demographics (BMI; SBP; DBP)

Model 3: adjusted for model 2 and comorbidities (renal function, CVD, PAD, diabetes, hypertension)

Model 4: adjusted for model 3 and proteinuria, use of anti RAAS agents

Statistical significance was defined as a p-value <0.05. Analyses were carried out with R version 3.3.2 (2016-10-31), the R Foundation for Statistical Computing for Mac.

Treatment with SB was associated with a significantly lower CKD progression irrespective of the multiple adjustment for confounders, further corroborating the results of the UBI study primary endpoint.

(C) Supplementary analysis to explore the potential effect of any of the main demographic and clinical characteristics on the effects of sodium bicarbonate on real function or all-cause mortality

To explore the potential effect of any of the main demographic and clinical characteristics on the effects of sodium bicarbonate on real function or all-cause mortality, Cox proportional-hazard model was utilized. Multiple augmented models are constructed each with an individual interaction of one covariate with the treatment effect.

Supplemental tables reports on the effects of the treatment within each category of each dichotomy or categorical variable and within ranges of each continuous variable.

We explored the potential effect modification of sodium bicarbonate supplementation on:

(i) creatinine doubling:

Condition	Hazard Ratio (HR)	95% Confidence Interval LowerLimit	UpperLimit	P-value	P interaction
Age <72 years	0.326	0.168	0.634	0.001	0.670
Age ≥72 years	0.406	0.212	0.778	0.007	
Female	0.394	0.199	0.777	0.007	0.765
Male	0.341	0.181	0.646	0.001	
SBP <130 mmHg	0.283	0.148	0.540	0.000	0.209
SBP ≥130 mmHg	0.506	0.256	0.999	0.050	
DBP <75 mmHg	0.388	0.202	0.744	0.004	0.755
DBP ≥75 mmHg	0.343	0.177	0.666	0.002	
No diabetes	0.405	0.233	0.702	0.001	0.503
Diabetes	0.289	0.122	0.688	0.005	
No hypertension	0.105	0.013	0.821	0.032	0.216
Hypertension	0.413	0.255	0.671	0.000	
No CV disease	0.295	0.171	0.509	0.000	0.077
CV disease	0.760	0.293	1.973	0.573	
Creatinine clearance					0.496
< 21 ml/min	0.168	0.057	0.499	0.001	
21-27 ml/min	0.203	0.069	0.591	0.003	
28-37 ml/min	0.753	0.287	1.980	0.566	
>37 ml/min	0.479	0.219	1.046	0.065	
Serum bicarbonate					0.324
<21 mmol/l	0.284	0.079	1.019	0.054	
21-22 mmol/l	0.310	0.134	0.716	0.006	
22-23 mmol/l	0.480	0.167	1.383	0.174	
>23 mmol/l	0.452	0.203	1.007	0.052	

Table legend: SBP systolic blood pressure; DBP diastolic blood pressure; CV cardiovascular

(ii) dialysis inception:

Condition	Hazard Ratio (HR)	95% Confidence Interval LowerLimit	UpperLimit	P-value	P interaction
Age <72 years	0.378	0.186	0.769	0.007	0.217
Age ≥72 years	0.672	0.344	1.313	0.245	
Female	0.879	0.395	1.957	0.752	0.105
Male	0.369	0.198	0.691	0.002	
SBP <130 mmHg	0.501	0.238	1.053	0.068	0.981
SBP ≥130 mmHg	0.510	0.270	0.962	0.038	
DBP <75 mmHg	0.336	0.167	0.676	0.002	0.104
DBP ≥75 mmHg	0.789	0.394	1.580	0.503	
No diabetes	0.336	0.178	0.633	0.001	**0.030**
Diabetes	1.090	0.466	2.552	0.842	
No hypertension	0.000	NA	NA	0.999	0.996*
Hypertension	0.244	0.067	0.886	0.032	
No CV disease	0.585	0.345	0.990	0.046	0.247
CV disease	0.244	0.067	0.886	0.032	
Creatinine clearance					0.341
< 21 ml/min	0.255	0.124	0.522	0.000	
21-27 ml/min	0.083	0.011	0.641	0.017	
28-37 ml/min	0.622	0.245	1.581	0.318	
>37 ml/min	0.428	0.083	2.207	0.311	
Serum bicarbonate					0.162
<21 mmol/l	0.255	0.124	0.522	0.000	
21-22 mmol/l	0.083	0.011	0.641	0.017	
22-23 mmol/l	0.622	0.245	1.581	0.318	
>23 mmol/l	0.428	0.083	2.207	0.311	

Table legend: SBP systolic blood pressure; DBP diastolic blood pressure; CV cardiovascular

*Only 2 events among non hypertensive subjects

(iii) all-cause mortality

Condition	Hazard Ratio (HR)	95% Confidence Interval LowerLimit	UpperLimit	P-value	P interaction
Age <72 years	0.427	0.128	1.421	0.165	0.985
Age ≥72 years	0.444	0.192	1.029	0.058	
Female	0.342	0.091	1.290	0.113	0.633
Male	0.486	0.216	1.091	0.080	
SBP <130 mmHg	0.518	0.217	1.236	0.138	0.521

SBP ≥130 mmHg	0.336	0.107	1.055	0.062	
DBP <75 mmHg	0.414	0.127	1.345	0.142	0.878
DBP ≥75 mmHg	0.454	0.194	1.062	0.069	
No diabetes	0.389	0.171	0.885	0.024	0.559
Diabetes	0.632	0.170	2.354	0.494	
No hypertension	0.000	NA	NA	0.999	0.995^
Hypertension	0.285	0.057	1.412	0.124	
No CV disease	0.491	0.228	1.056	0.069	0.584
CV disease	0.285	0.057	1.412	0.124	
Creatinine clearance					0.147
< 21 ml/min	0.111	0.029	0.426	0.001	
21-27 ml/min	0.468	0.144	1.523	0.207	
28-37 ml/min	0.447	0.087	2.306	0.336	
>37 ml/min	1.139	0.230	5.645	0.873	
Serum bicarbonate					0.494*
<21 mmol/l	1.645	0.275	9.850	0.586	
21-22 mmol/l	0.420	0.111	1.585	0.200	
22-23 mmol/l	0.000	NA	NA	0.999	
>23 mmol/l	0.375	0.139	1.017	0.054	

Table legend: SBP systolic blood pressure; DBP diastolic blood pressure; CV cardiovascular

*Only 5 and 4 events among subjects with serum bicarbonate<21mmol/l and 22-23 mmol/l

^Only 4 events among hypertensive patients

(iv) the composite of all three endpoints.

Condition	Hazard Ratio (HR)	95% Confidence Interval Lower Limit	Upper Limit	P-value	P interaction
Age <72 years	0.357	0.228	0.559	0.000	0.670
Age ≥72 years	0.495	0.330	0.743	0.001	
Female	0.379	0.257	0.560	0.005	0.375
Male	0.509	0.317	0.817	0.000	
SBP <130 mmHg	0.386	0.253	0.588	0.000	0.504
SBP ≥130 mmHg	0.475	0.309	0.729	0.001	
DBP <75 mmHg	0.367	0.236	0.571	0.000	0.407
DBP ≥75 mmHg	0.487	0.323	0.734	0.001	
No diabetes	0.375	0.258	0.543	0.000	0.229
Diabetes	0.561	0.331	0.949	0.031	
No hypertension	0.070	0.009	0.526	0.010	0.059

Hypertension	0.432	0.221	0.846	0.014	
No CV disease	0.424	0.303	0.593	0.000	0.854
CV disease	0.432	0.221	0.846	0.014	
Creatinine clearance					0.295
< 21 ml/min	0.192	0.112	0.330	0.000	
21-27 ml/min	0.222	0.108	0.457	0.000	
28-37 ml/min	0.638	0.344	1.184	0.154	
>37 ml/min	0.537	0.284	1.016	0.056	
Serum bicarbonate					0.692
<21 mmol/l	0.604	0.287	1.269	0.183	
21-22 mmol/l	0.427	0.244	0.749	0.003	
22-23 mmol/l	0.420	0.202	0.875	0.021	
>23 mmol/l	0.369	0.226	0.602	0.000	

Table legend: SBP systolic blood pressure; DBP diastolic blood pressure; CV cardiovascular

Statistical significance was defined as a p-value <0.05. Analyses were carried out with R version 3.3.2 (2016-10-31), the R Foundation for Statistical Computing for Mac.
